# Supplementary material for: Assessment of hypertension and other factors associated with the severity of disease in COVID-19 pneumonia, Addis Ababa, Ethiopia: A case-control study
Source: PLoS One. 2022 Aug 15;17(8):e0273012. doi: 10.1371/journal.pone.0273012 (PMC9377616; doi:10.1371/journal.pone.0273012)
Supplement: Attachment — Submitted filename: Response letter 2 to PLOS ONE.docx [file pone.0273012.s002.docx]

PLOS ONE

June 22, 2022

Dear Editor,

We greatly appreciate the comments of the reviewers of our manuscript and have addressed them here, point by point, and revised our manuscript accordingly.

**Reviewer #1:** Work on data presentation and in-depth analysis of data alongside an expansion of the cohort size to meet the publication quality of the journal

**Response:** Thank you for the comments and concerns raised to perform deep analysis using Cox proportion hazard (CoH). Here, I will try to kindly respond to why we couldn’t conduct the analysis further. As you may recall, our study design was a case-control where recruitment of study subjects was based on the severity of disease (COVID-19) status. Cases were those with severe forms of the disease including critical, while controls were those who had mild to moderate disease, including asymptomatic ones. Some of the cases were recruited when they came to the hospital in a severe or critical state, while the rest were recruited after they developed severe disease. The exposure was hypertension whereas other related factors were considered possible confounders to suppress the effect of the main exposure(hypertension). Most importantly, from the nature of our retrospective data, the time between the onset of the disease and the change in severity course is difficult to retrieve and estimate. Thus, I respectfully would like to notify you that the design of our study will not allow us to use the CoH.

Concerning the sample size, we edited the sentence under the strength and limitation part, under the discussion section, where we tried to clarify for you the points we raised about the disparity between the initial calculated sample size (252, composed of 63 cases and 189 controls) and the actual number of patients (265, composed of 75 cases and 190 controls) with majority matched 1 to 3. So, here we acknowledge the limitation we faced due to the strict matching criteria of the design, and we were short of controls matching to cases for age bands, sex, and discharge date criteria though we had an extra number of cases (75 vs 63).

**In the paragraph on Strength and limitation part of the Discussion section**

However, this study had a few limitations. First, the strict matching criteria, including the date of discharge on selecting one case for three controls, failed to fulfill the planned **case-to-control ratio**, we collected data for 75 cases for which we were supposed to collect proportionally 225 controls which makes the total sample size to be 300. However, we **were able to retrieve data** **of** 75 cases for 190 **controls** which made the total sample size **265**, **which is a bit higher than the initial calculated sample size (252, 63 cases for 189 controls) with a slightly higher number of those on case arm (75 vs 63)**.

Thank you for considering our research article. We hope that our revisions meet with your approval.

With best regards,
